# Activity of a Synthetic Peptide Targeting MgtC on *Pseudomonas aeruginosa* Intramacrophage Survival and Biofilm Formation

**DOI:** 10.3389/fcimb.2019.00084

**Published:** 2019-04-02

**Authors:** Malika Moussouni, Pauline Nogaret, Preeti Garai, Bérengère Ize, Eric Vivès, Anne-Béatrice Blanc-Potard

**Affiliations:** ^1^Laboratoire de Dynamique des Interactions Membranaires Normales et Pathologiques, Université Montpellier, Montpellier, France; ^2^CNRS, UMR5235, Montpellier, France; ^3^Laboratoire d'Ingénierie des Systèmes Macromoléculaires, Institut de Microbiologie de la Méditerranée, CNRS & Aix-Marseille University of Marseille, Marseille, France; ^4^Centre de Recherche en Biologie cellulaire de Montpellier, CNRS UMR 5237, Montpellier, France

**Keywords:** *Pseudomonas aeruginosa*, antivirulence strategy, MgtC, peptide, macrophage, EPS

## Abstract

Antivirulence strategies aim to target pathogenicity factors while bypassing the pressure on the bacterium to develop resistance. The MgtC membrane protein has been proposed as an attractive target that is involved in the ability of several major bacterial pathogens, including *Pseudomonas aeruginosa*, to survive inside macrophages. In liquid culture, *P. aeruginosa* MgtC acts negatively on biofilm formation. However, a putative link between these two functions of MgtC in *P. aeruginosa* has not been experimentally addressed. In the present study, we first investigated the contribution of exopolysaccharides (EPS) in the intramacrophage survival defect and biofilm increase of *mgtC* mutant. Within infected macrophages, expression of EPS genes *psl* and *alg* was increased in a *P. aeruginosa mgtC* mutant strain comparatively to wild-type strain. However, the intramacrophage survival defect of *mgtC* mutant was not rescued upon introduction of *psl* or *alg* mutation, suggesting that MgtC intramacrophage role is unrelated to EPS production, whereas the increased biofilm formation of *mgtC* mutant was partially suppressed by introduction of *psl* mutation. We aimed to develop an antivirulence strategy targeting MgtC, by taking advantage of a natural antagonistic peptide, MgtR. Heterologous expression of *mgtR* in *P. aeruginosa* PAO1 was shown to reduce its ability to survive within macrophages. We investigated for the first time the biological effect of a synthetic MgtR peptide on *P. aeruginosa*. Exogenously added synthetic MgtR peptide lowered the intramacrophage survival of wild-type *P. aeruginosa* PAO1, thus mimicking the phenotype of an *mgtC* mutant as well as the effect of endogenously produced MgtR peptide. In correlation with this finding, addition of MgtR peptide to bacterial culture strongly reduced MgtC protein level, without reducing bacterial growth or viability, thus differing from classical antimicrobial peptides. On the other hand, the addition of exogenous MgtR peptide did not affect significantly biofilm formation, indicating an action toward EPS-independent phenotype rather than EPS-related phenotype. Cumulatively, our results show an antivirulence action of synthetic MgtR peptide, which may be more potent against acute infection, and provide a proof of concept for further exploitation of anti-*Pseudomonas* strategies.

## Introduction

The increasing understanding of bacterial pathogenesis has revealed potential strategies to develop novel drugs against infectious bacteria (Heras et al., 2015; Hauser et al., 2016; Mühlen and Dersch, 2016; Dickey et al., 2017). Interference with bacterial virulence is a promising alternative approach or a complementary adjunct to traditional antimicrobial therapy. This approach does not mediate direct bacterial killing, at least *in vitro*, therefore it is thought to apply less selective pressure for resistance and better preserve microbiota. The MgtC membrane protein has been proposed as a suitable target for antivirulence strategies because it is a virulence factor conserved in several bacterial pathogens (Alix and Blanc-Potard, 2007; Belon and Blanc-Potard, 2016). MgtC, which was first described in *Salmonella enterica* serovar Typhimurium (*S*. Typhimurium), is a critical factor for the intramacrophage survival of various unrelated intracellular pathogens (*Salmonella* spp., *Mycobacterium tuberculosis, Brucella suis* and *Burkholderia cenocepacia*), as well as so-called extracellular pathogens that can transiently reside within cells (*Yersinia pestis, Pseudomonas aeruginosa*) (Blanc-Potard and Groisman, 1997; Buchmeier et al., 2000; Lavigne et al., 2005; Grabenstein et al., 2006; Maloney and Valvano, 2006; Belon et al., 2015).

The environmental bacterium and opportunistic human pathogen *P. aeruginosa* is responsible for a variety of acute infections and is a major cause of mortality in chronically infected cystic fibrosis (CF) patients. Due to the increasing number of antibiotic resistant clinical isolates, *P. aeruginosa* has been listed in the WHO top priority list for drug development (Tacconelli et al., 2018). The chronic infection of *P. aeruginosa* and its resistance to treatment is largely due to its ability to form biofilms, which relies on the production of exopolysaccharides (EPS), as reviewed by Klockgether and Tümmler (2017). Recent studies have highlighted the role of intracellular stages during *P. aeruginosa* infection, which may contribute to bacterial dissemination and *in vivo* resistance to antibiotics (Brannon et al., 2009; Buyck et al., 2013). In *P. aeruginosa, mgtC* expression is induced inside macrophages, which is consistent with its contribution to bacterial intramacrophage survival, and the *mgtC* mutant (in the PAO1 background) is attenuated in an acute model of infection in zebrafish embryos (Belon et al., 2015; Bernut et al., 2015). MgtC was also found to limit biofilm formation and EPS production in magnesium deprived medium, a condition known to activate *mgtC* transcription (Belon et al., 2015). Interestingly, MgtC has been shown to repress cellulose production in *S*. Typhimurium, which in turn promotes bacterial replication within macrophages (Pontes et al., 2015). Cellulose is not produced by *P. aeruginosa*, but these results suggested a potential link between the production of *P. aeruginosa* EPS and intramacrophage behavior (Belon et al., 2015; Bernut et al., 2015). Three polysaccharides, namely Psl, Pel and alginate, compose *P. aeruginosa* EPS (Wei and Ma, 2013), and contribute to the formation of biofilms (Ghafoor et al., 2011). Psl has a major contribution in biofilm formation, being required for adhesion of bacteria, which is critical for biofilm initiation, as well as maintenance of the biofilm structure. Pel is responsible for pellicle formation on air-liquid interface and formation of solid surface-associated biofilms. Pel could function together with other EPS throughout biofilm development in *P. aeruginosa* PAO1, although in a less important way as compared to Psl (Yang et al., 2011). Alginate is the exopolysaccharide that is mainly produced by *P. aeruginosa* clinical isolates from the lungs of CF patients, where it plays important roles in structural stability and protection of biofilms. However, it is not absolutely required during the formation of non-mucoid biofilms *in vitro*, as shown for PAO1 strain (Wozniak et al., 2003)

Of interest in the context of an antivirulence approach, a natural peptide antagonistic to MgtC has been described. This peptide, called MgtR, is a very small membrane protein of 30 amino-acids (thus referred as peptide) identified in *S*. Typhimurium, where it promotes the degradation of the MgtC virulence factor by FtsH protease (Alix and Blanc-Potard, 2008; Lee and Groisman, 2010). MgtR is part of a novel class of regulatory molecules that can interact with membrane proteins and can subsequently act on the stability of these membrane proteins (Alix and Blanc-Potard, 2009; Lippa and Goulian, 2009; Wang et al., 2017). Overexpression of *mgtR* reduced intramacrophage survival of a wild-type *Salmonella* strain, thus indicating that MgtR can lower *Salmonella* virulence (Alix and Blanc-Potard, 2008). MgtR has been shown to directly interact with the *Salmonella* MgtC membrane protein (Alix and Blanc-Potard, 2008). Moreover, the *Salmonella* MgtR membrane peptide can also interact with MgtC protein from *M. tuberculosis* and *P. aeruginosa* (Belon et al., 2015, 2016). *M. tuberculosis* and all *P. aeruginosa* strains naturally lack *mgtR* gene, but ectopic expression of *Salmonella mgtR* in mycobacteria and *P. aeruginosa* mimicked the phenotypes reported for *mgtC* deletion mutants (Belon et al., 2015, 2016). Thus, these findings show an antivirulence action of MgtR upon endogenous production in several bacterial pathogens.

In the present study, we addressed the contribution of EPS to the phenotypes of *P. aeruginosa mgtC* mutant and investigated further an antivirulence strategy targeting MgtC on *P. aeruginosa*. We first investigated the contribution of individual EPS components (alginate, Psl and Pel) to the intramacrophage survival defect of *P. aeruginosa mgtC* mutant as well as biofilm formation. Then, studies with synthetic peptide added exogenously were carried out for the first time to further evaluate the efficiency of an antivirulence strategy based on MgtR. We investigated the biological effect of a synthetic MgtR peptide toward *P. aeruginosa* ability to survive inside macrophages and form biofilm.

## Materials and Methods

### Bacterial Strains and Growth Conditions

Bacterial strains and plasmids are described in Table 1. *P. aeruginosa* strains are in the PAO1 background. *P. aeruginosa* was grown at 37°C in Luria broth (LB). Growth in magnesium-deprived medium was done in No-carbon essential (NCE)-minimal medium (28 mM KH_2_PO_4_, 28 mM K_2_HPO_4_·3H_2_O, 16 mM NaNH_4_HPO_4_·4H_2_O) supplemented with 0.1% casamino acids, 38 mM glycerol and 10 μM MgSO_4_ as described earlier (Rang et al., 2007). Plasmid pMF230 (constitutive GFP expression, obtained from Addgene) was introduced in *P. aeruginosa* by conjugation, using an *E. coli* strain containing pRK2013. Recombinant bacteria were selected on *Pseudomonas* isolation agar (PIA) containing carbenicillin at the concentration of 300 μg/ml. Construction of *mgtC psl* and *mgtC alg* double mutant strains was done as described earlier (Belon et al., 2015) by allelic exchange of the *pslD* gene in *mgtC* mutant using a pKNG*pslD* suicide plasmid or allelic exchange of the *mgtC* gene in the *alg8* mutant (harboring a deletion of *alg* operon) using the pSBC44 suicide plasmid, respectively (Table 1).

**Table 1:** Bacterial strains and plasmids used in the study.

**Name**	**Description**	**References**
PAO1	Wild-type	Laboratory collection
PAO1 Δ*mgtC*		Belon et al., 2015
PAO1 Δ*alg8*	*alg8* deletion mutant	Periasamy et al., 2015
PAO1 Δ*mgtC* Δ*psl*	Δ*mgtC* Δ*pslD*	This study
PAO1 Δ*mgtC* Δ*alg*	Δ*mgtC* Δ*alg8*	This study
pRK2013	Tra^+^, Mob^+^, ColE1, KmR	Laboratory collection
pMF230	GFP*mut2*	Nivens et al., 2001
pKNG101	Suicide vector Sm^R^; *sacB^+^*	Kaniga et al., 1991
pKNG*pslD*	Δ*pslD* deletion in pKNG101	This study
pSBC44	Δ*mgtC* deletion in pKNG101	Belon et al., 2015

### Peptide Synthesis

MgtR peptide (MNRSPDKIIALIFLLISLLVLCLALWQIVF) was synthesized by a solid-phase method using the Fmoc methodology on an automated microwave peptide synthesizer (Liberty-1, CEM, Orsay, France) as previously described (Belon et al., 2016). H-Rink amide ChemMatrix resin (100 micromoles, substitution 0.37 mmol/g, Longjumeau, France) was used. The peptide was synthesized following a double-coupling step for each amino acid (400 micromoles) activated with TBTU (500 micromoles). Additionally an acetylation step was applied at the end of each amino-acid incorporation to prevent the formation of incomplete peptides. At the end of the assembly, the N-terminal Fmoc was kept on the peptide and the peptide/resin was stored at 4°C under inert atmosphere. When required, peptide/resin was treated to remove the Fmoc group, rinsed and dried under vacuum before the final deprotection procedure. The peptide/resin was then treated with 10 ml/g of cleavage cocktail (trifluoroacetic acid/triisopropylsilane/H_2_O/1,2-ethanedithiol (94/1/2.5/2.5%: vol/vol) at room temperature for 90 min. The peptide was then precipitated with cold ether and centrifuged before resuspension in acetonitrile/water (30/70: vol/vol), freezing and freeze-drying. The final product was resuspended in isopropanol/water (50%/50%: vol/vol) without any purification and analyzed by MALDI-TOF mass spectrometry. The expected mass is 3,456.39 and the measured one was 3,456.78 ([MH]^+^). Circular dichroism spectra were recorded on a Jasco 810 (Japan) dichrograph in quartz suprasil cells (Hellma) with an optical path of 1 mm using 40 μM peptide in 30% isopropanol with and without SDS 4%. For experimentations, peptide was resuspended at a concentration of 3.2 mM in DMSO/water (50%/50%: vol/vol) and sonicated for 15 min before use. Using the same procedure, we also synthesized a scrambled peptide based on the amino-acid sequence of MgtR (permutation of the original peptide, ILFVADSLQMIPLCLRIWVALKINILFSVL). The measured mass correlated with the expected one.

### Infection of Macrophages and Quantification of Intracellular Bacteria

J774 cells (J774A.1) were maintained at 37°C in 5% CO_2_ in Dulbecco's modified Eagle medium (DMEM) (Gibco) supplemented with 10% fetal bovine serum (FBS) (Gibco). The infection of J774 macrophages by *P. aeruginosa* was carried out essentially as described previously (Belon et al., 2015). Mid-log phase *P. aeruginosa* grown in LB broth was centrifuged and resuspended in PBS to infect J774 macrophages (5×10^5^ cells/well) at an MOI of 10. After 5 min centrifugation of the 24-well culture plate for synchronization of infection, bacterial phagocytosis was allowed to proceed for 25 min. Cells were washed three times with sterile PBS and fresh DMEM media supplemented with 400 μg/ml gentamicin was added, which was retained throughout the infection. Macrophages were lysed after 20 min (T0) or 2 h (T1) of gentamicin treatment, by using 0.1% Triton X-100 and the number of viable bacteria was determined by subsequent plating onto LB agar plates. The percentage of survival was obtained by multiplying with 100 the ratio of the bacterial colony forming units (CFU) at T1 to that of T0.

### RNA Extraction and Quantitative RT-PCR (qRT-PCR)

For bacterial RNA extraction from infected J774, 6.5 × 10^6^ macrophages were seeded into a 100 cm^2^ tissue culture dish and infected at an MOI of 10 as described above. One hour after phagocytosis, cells were washed three times with PBS, lysed with 0.1% Triton X100 and pelleted by centrifugation at 13,000 rpm for 10 min at 15°C. Bacteria were resuspended in 500 μl PBS and the non-resuspended cellular debris were discarded. 900 μl of RNA protect reagent (Qiagen) was added and incubated for 5 min. The sample was centrifuged at 13,000 rpm for 10 min. Bacteria in the pellet were lysed with lysozyme and RNA was prepared with RNEasy kit (Qiagen). Superscript III reverse transcriptase (Invitrogen) was used for reverse transcription. Controls without reverse transcriptase were done on each RNA sample to rule out possible DNA contamination. Quantitative real-time PCR (Q-RT-PCR) was performed using a Light Cycler 480 SYBR Green I Master mix in a 480 Light Cycler instrument (Roche). PCR conditions were as follows: 3 min denaturation at 98°C, 45 cycles of 98°C for 5 s, 60°C for 10 s, and 72°C for 10 s. The sequences of primers used for RT-PCR are listed in Supplemental Table 1.

### LDH Assay

The cytotoxicity of synthetic peptide was assessed by release of lactate dehydrogenase (LDH) using the Pierce LDH cytotoxicity assay kit (Thermo Scientific). Macrophages were seeded in a 96 well plate and treated with peptide (120 μM) for 18 h. The assay was performed on 50 μl of the culture supernatant according to manufacturer's instructions. The percentage of LDH release was first normalized to that of the untreated control and then calculated relatively to that of untreated cells lysed with Triton X-100, which was set at 100% LDH release.

### Biofilm Formation

Biofilm formation in LB medium was carried out in 96-well plate. Overnight culture of PAO1, grown in LB, was diluted 1:100 in LB and 200 μl was aliquoted in 96-well plate in triplicates. Peptide was added at the concentration of 120 μM and DMSO (2% final) was used as solvent control. The plate was incubated at 28°C for 48 h under static condition. After 48 h, culture was discarded and wells were washed with water. Biofilm appeared in the form of rings on the walls of the wells. Crystal Violet (CV) was added to each well to stain the biofilm for 15 min at room temperature. After staining, wells were washed with water to remove excess of CV and 70% ethanol was added to dissolve the stain. The dissolved stain was taken in a fresh plate to measure the absorbance at 570 nm.

Biofilm formation in low magnesium medium was carried out in glass tubes. Overnight culture of bacterial strains, grown in LB, was washed with NCE medium and diluted 1:20 into 500 μl of NCE medium supplemented with 10 μM Mg^2+^ in the glass tube. MgtR peptide was added at the concentration of 120 μM and DMSO was used as solvent control. The glass tubes were incubated at 30°C for 24 h under static condition. Staining of biofilm ring with CV (0.1%) was quantified as described above. To measure bacterial viability, aliquots of bacterial culture and PBS-washed ring were diluted in PBS for CFU counting.

### Preparation of Lysates From Bacterial Cultures Treated With Synthetic Peptide

Overnight culture of *P. aeruginosa* grown in LB medium was diluted 1:5 in LB and grown until OD_600nm_ of 0.8 to 1. Bacteria were washed in NCE medium (Blanc-Potard and Groisman, 1997) without magnesium and were resuspended in the same medium supplemented with 10 μM Mg^2+^ and synthetic peptides (at the indicated concentration) or DMSO as control. Bacteria were harvested after 4–5 h of incubation at 37°C. To prepare whole-cell extracts, cultures were normalized to OD_600nm_, centrifuged, re-suspended in Laemmli buffer and lysed by heating at 95°C for 10 min before performing Western Blot assay.

### Preparation of Anti-PA4635 Antibodies

A DNA fragment encoding the C-terminal domain of MgtC (PA4635) was amplified using primers indicated in Supplemental Table 1 and cloned into pQE30 vector (Qiagen). Recombinant plasmid was introduced into M15/pRep4 *E. coli* strain to express and purify the His-tagged recombinant protein as described (Alix and Blanc-Potard, 2008). Polyclonal antibodies were produced upon immunization of rabbit with the PA4635 C-ter domain (Eurogentec).

### Western Blot Analysis

Total bacterial lysates were electrophoresed on 12.5% SDS-PAGE gels and transferred onto nitrocellulose membrane (Invitrogen) for immunoblotting. Rabbit anti-PA4635 were used at 1:2,000 dilution and Mouse anti-EF-Tu antibody (Ball et al., 2016) was used at 1:20,000 dilution. Anti-rabbit or anti-mouse antibodies (dilution 1:30,000) labeled with fluorescent dye DyLight 800 (Thermoscientific) were used for the detection of PA4635 and EF-Tu using LICOR Odyssey Fc Imaging System (excitation 783 nm and emission 797 nm) to quantify the amount of proteins.

## Results and Discussion

### Contribution of EPS to the Intramacrophage Survival Defect and Biofilm Phenotype of *P. aeruginosa*

**Figure 1:**
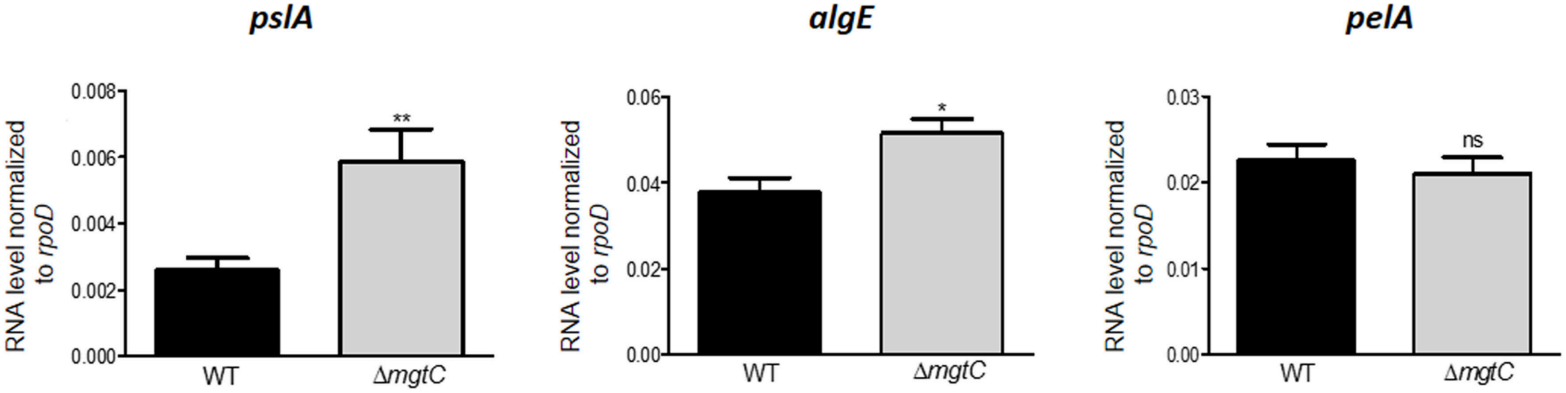
Expression of EPS genes in *P. aeruginosa* strains residing in J774 macrophages. Expression of EPS genes in strains PAO1 wild-type (WT) and Δ*mgtC* mutant infecting J774 macrophages. RNA was extracted from bacteria isolated from infected macrophages 1 h after phagocytosis. The level of *pslA, algE*, and *pelA* transcripts relative to those of the house-keeping gene *rpoD* was measured by qRT-PCR and plotted on the Y-axis. Error bars correspond to standard errors from at least three independent experiments. The asterisks indicate *P*-values (Student's t-test *P < 0.05, **P < 0.01), showing statistical significance with respect to WT.

*In vitro, P. aeruginosa* PAO1 produces mainly Psl and Pel, whereas alginate synthesis is negatively regulated in this non-mucoidal strain by the anti-sigma factor of MucA (Schurr et al., 1996; Franklin et al., 2011). However, the expression of EPS genes has not been measured when PAO1 resides in macrophages. To address a putative link between the intramacrophage survival defect of *P. aeruginosa mgtC* mutant and EPS production, we first analyzed the level of expression of candidate individual EPS genes, *pelA, pslA*, and *algE*, in *P. aeruginosa mgtC* mutant and wild-type strain residing in J774 macrophages. Each of these genes is a part of the respective operons required for synthesis of Pel, Psl and alginate polysaccharides (Franklin et al., 2011). A significant increased expression of *pslA* and, to a lesser extent, *algE* was detected in *mgtC* mutant as compared to wild-type strain, whereas *pelA* expression remained unaltered (Figure 1), suggesting a higher production of Psl and alginate inside macrophages by *mgtC* mutant.

**Figure 2:**
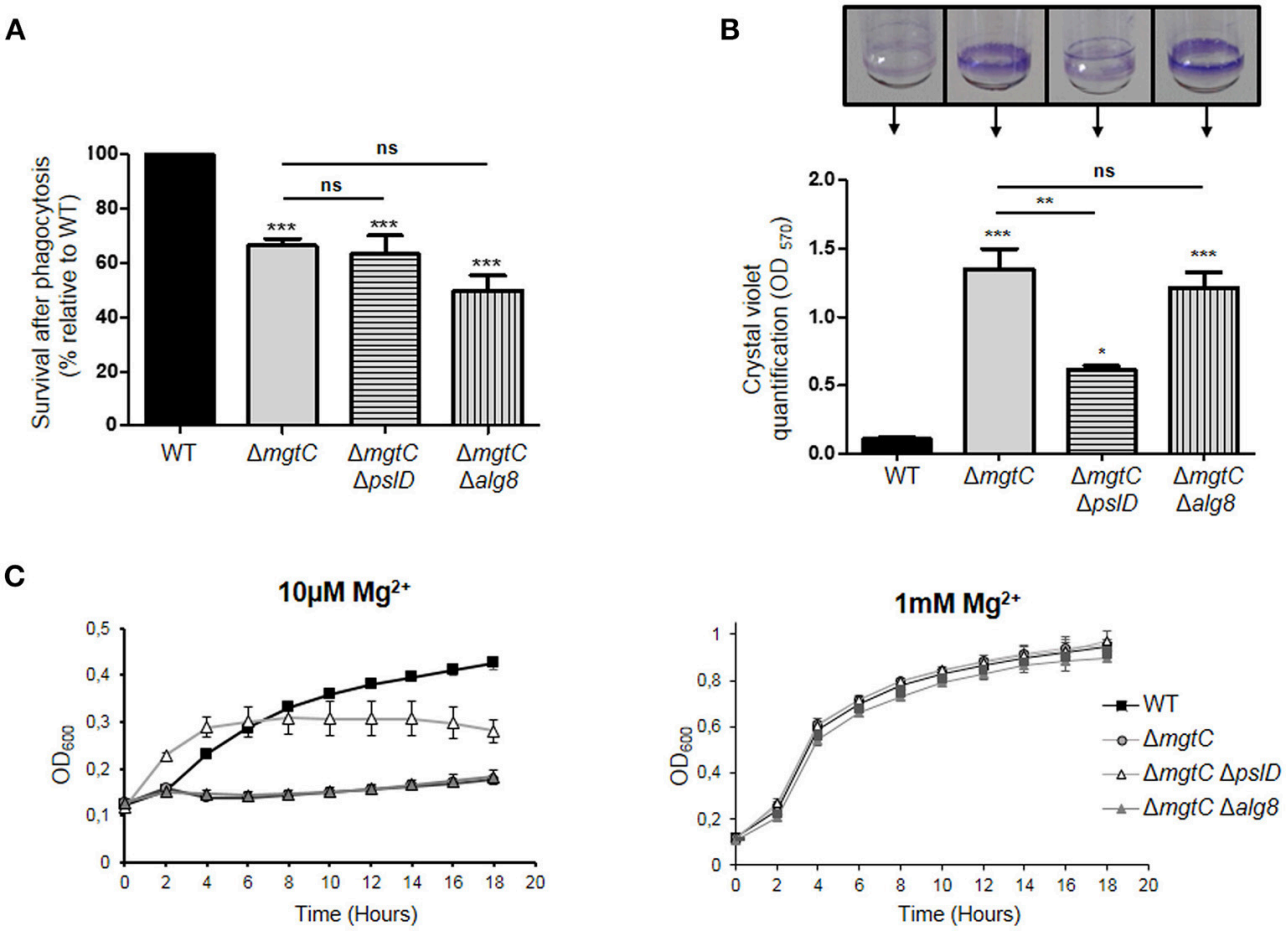
Phenotypes of *mgtC pslD* and *mgtC alg8* double mutants. **(A)** Behavior of *mgtC pslD* and *mgtC alg8* double mutant within J774 macrophages. Survival of *P. aeruginosa* PAO1 WT, Δ*mgtC*, Δ*mgtC* Δ*pslD* and Δ*mgtC* Δ*alg8* strains was assessed upon phagocytosis by J774 macrophages. Results are expressed as percentage of surviving bacteria 2 h after phagocytosis compared to the number of bacteria 20 min after phagocytosis and are normalized to 100% for WT strain. Error bars correspond to standard errors from four independent experiments. The asterisks indicate *P*-values (One-way ANOVA followed by a Bonferroni post-test. ***P < 0.001). **(B)** Quantification of bacterial adherence to glass tubes to infer the ability of strains to form biofilm. *P*. *aeruginosa* strains PAO1 WT, Δ*mgtC*, Δ*mgtC* Δ*pslD*, and Δ*mgtC* Δ*alg8* were grown at 30°C for 24 h in NCE medium with 10 μM MgSO_4_. The biofilm quantification is visualized by CV ring on the glass tubes and CV is quantified at 570 nm. Error bars correspond to standard errors (+SE) from three independent experiments and the asterisks indicate *P*-values (One-way ANOVA followed by a Bonferroni post-test, *P < 0.05, **P < 0.01, ***P < 0.001). **(C)** Growth curve of *P*. *aeruginosa* strains PAO1, Δ*mgtC*, Δ*mgtC* Δ*pslD* and Δ*mgtC* Δ*alg8* in NCE medium supplemented with 10 μM (left panel) or 1 mM (right panel) MgSO_4_. The results are expressed as the mean ± SD of three independent experiments.

A *Salmonella mgtC* mutant has been reported to exhibit increased production of cellulose, another type of exopolysaccharide, inside macrophages, and the intramacrophage replication defect of the *Salmonella mgtC* mutant could be reversed by an additional mutation in the *bcsA* gene that prevented cellulose synthesis (Pontes et al., 2015). To investigate whether the intramacrophage survival defect of the *P. aeruginosa mgtC* mutant could be related to increased Psl or alginate production within macrophages, we constructed *mgtC pslD*, and *mgtC alg8* double mutants (see Materials and Methods), because *pslD* and *alg8* are essential for Psl and alginate synthesis, respectively (Remminghorst and Rehm, 2006; Byrd et al., 2009). No rescue in the survival inside macrophages was observed for *mgtC* mutant in the presence of *psl* or *alg* mutation (Figure 2A), indicating that the survival defect of *P. aeruginosa mgtC* mutant is not linked to increased production of Psl or alginate. Thus, despite the observed increased expression of *psl* and *alg* genes, EPS did not contribute to the intramacrophage phenotype of *P. aeruginosa mgtC* mutant. These findings differ from the results obtained in *S*. Typhimurium with *mgtC bcsA* double mutant (Pontes et al., 2015). In contrast to *P. aeruginosa, Salmonella* actively replicates inside macrophages, and increased cellulose production may somehow contribute to the replication defect of *Salmonella mgtC* mutant within macrophages.

As reported earlier, the formation of biofilm was found to be significantly increased for *mgtC* mutant comparatively to wild-type strain in low magnesium medium (Figure 2B), a condition that induces expression of *mgtC* gene (Belon et al., 2015). The formation of biofilm of *mgtC alg8* double mutant was identical to the one of *mgtC* mutant, whereas the biofilm formation was significantly reduced in *mgtC pslD* double mutant comparatively to *mgtC* mutant (Figure 2B). This finding correlated with the growth curves of the different strains in low magnesium (10 μM) medium (Figure 2C), where growth of *mgtC* mutant was impaired in low magnesium medium comparatively to wild-type strain, and presence of a *pslD* mutation, but not an *alg8* mutation, improved the growth of the *mgtC* mutant (Figure 2C). All strains grew similarly in medium supplemented with 1 mM magnesium (Figure 2C).

Non-mucoid *P. aeruginosa* strains, as PAO1, utilize primarily the Psl and Pel polysaccharides for biofilm formation, whereas alginate is the predominant extracellular polysaccharide of the matrix of mucoid strains (Wozniak et al., 2003). Our results indicate that Psl polysaccharide, which is known to be more important than Pel polysaccharide in *P. aeruginosa* PAO1 biofilm formation (Yang et al., 2011), contributes to the increased biofilm formation and impaired growth of *mgtC* mutant in magnesium deprived medium. The growth defect of the *mgtC* mutant in low magnesium medium is associated with the formation of bacterial aggregates both on the tube (at the air-liquid level) and within the culture, thus reminiscent of biofilm initiation. This is consistent with the contribution of Psl, which is known to be produced during planktonic growth, mediating attachment to surfaces and contributing to microcolony formation (Ghafoor et al., 2011).

### Effect of Synthetic MgtR Peptide on Macrophage Survival of Wild-Type *P. aeruginosa* PAO1 Strain

**Figure 3:**
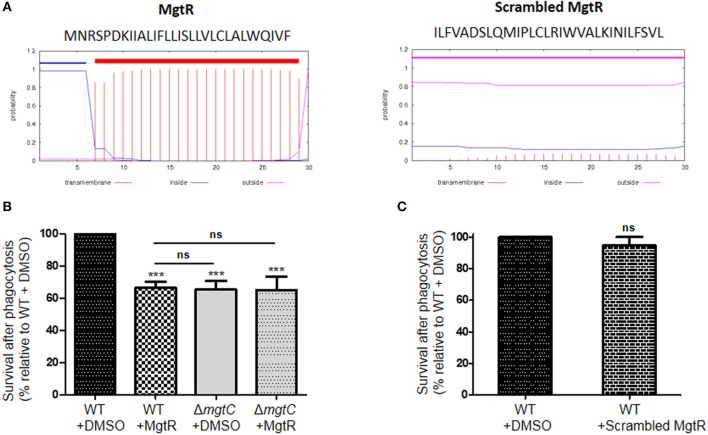
Effect of synthetic MgtR peptide on *P*. *aeruginosa* intramacrophage survival. **(A)** Sequence and transmembrane helix prediction for MgtR and scrambled MgtR peptides, using TMHMM program (http://www.cbs.dtu.dk/services/TMHMM/). **(B)** To test the effect of synthetic MgtR peptide on *P*. *aeruginosa* intramacrophage survival, PAO1 WT strain was treated with 120 μM of MgtR peptide or DMSO for 15 min before phagocytosis. A Δ*mgtC* mutant treated with DMSO or MgtR peptide (120 μM) is used as control. Error bars correspond to standard errors (+SE) from at least four experiments. Statistical analysis was performed with one-way ANOVA followed by a Bonferroni post-test, ***P < 0.001. **(C)** To test the effect of synthetic scrambled MgtR peptide on *P*. *aeruginosa* intramacrophage survival, PAO1 WT strain was treated with 120 μM of MgtR scrambled peptide or DMSO for 15 min before phagocytosis. Error bar corresponds to standard errors (+SE) from at least three experiments. Statistical analysis was performed with one-way ANOVA followed by a Bonferroni post-test.

*P. aeruginosa* naturally lacks *mgtR* gene, but ectopic expression of *Salmonella mgtR* in *P. aeruginosa* reduced intramacrophage survival, thus mimicking the phenotype reported for *mgtC* deletion mutant (Belon et al., 2015). To investigate the effect of exogenous addition of peptide, we added synthetic MgtR peptide to *P. aeruginosa*. The MgtR peptide, which harbors 73% of hydrophobic residues, is predicted to be organized in a single transmembrane helix (Figure 3A), which has been confirmed by an NMR structural analysis (Jean-Francois et al., 2014). MgtR was chemically synthesized using optimized protocol for hydrophobic peptides (see Materials and Methods) and the alpha-helical fold was verified by circular dichroism (Supplemental Figure S1). To address the effect of synthetic peptide on the ability of wild-type *P. aeruginosa* PAO1 strain to survive inside macrophages, bacteria were treated for 15 min, before phagocytosis, with 120 μM of peptide solubilized in DMSO. Bacteria pretreated with DMSO were used as control. Phagocytosis by J774 macrophages was allowed for 30 min before removing extracellular bacteria with PBS washes and gentamicin treatment. The bacterial survival was then monitored for 2 h after phagocytosis. Bacteria treated with MgtR peptide had a significantly lower intramacrophage survival than bacteria treated with DMSO, which is in the same range as the one observed for *mgtC* mutant (Figure 3B). Interestingly, addition of MgtR peptide did not lower the survival of *mgtC* mutant, suggesting that the presence of MgtC plays a role in the biological activity of the synthetic peptide toward macrophage infection (Figure 3B). In the condition used, the MgtR peptide is not cytotoxic to J774 cells as shown by quantification of LDH release (Supplemental Figure S2). We designed and synthesized a scrambled peptide that contained the amino-acids of MgtR in a scrambled order, thus retaining the hydrophobicity, but lacking a predicted membrane helix, as shown using the TMHMM program (Figure 3A). As expected, the scrambled peptide exhibited a circular dichroism spectra that differs from the one of MgtR, indicative of a loss of the alpha-helix structure (Supplemental Figure S1). Importantly, no significant decrease of intramacrophage survival was observed with this scrambled peptide used at 120 μM (Figure 3C), suggesting that the biological effect of the synthetic MgtR peptide is somehow linked to sequence specificity and not only to overall hydrophobicity.

Intramacrophage survival assay with wild-type *P. aeruginosa* strain showed that addition of exogenous MgtR peptide mimics the phenotype of *mgtC* mutant as well as the effect previously reported upon endogenous expression of the peptide in PAO1, supporting a biological activity exhibited by this synthetic peptide. Moreover, this biological activity is linked to the presence of MgtC.

### Effect of Synthetic MgtR Peptide on MgtC Protein Level, Bacterial Growth and Biofilm Formation With *P. aeruginosa* Wild-Type PAO1

**Figure 4:**
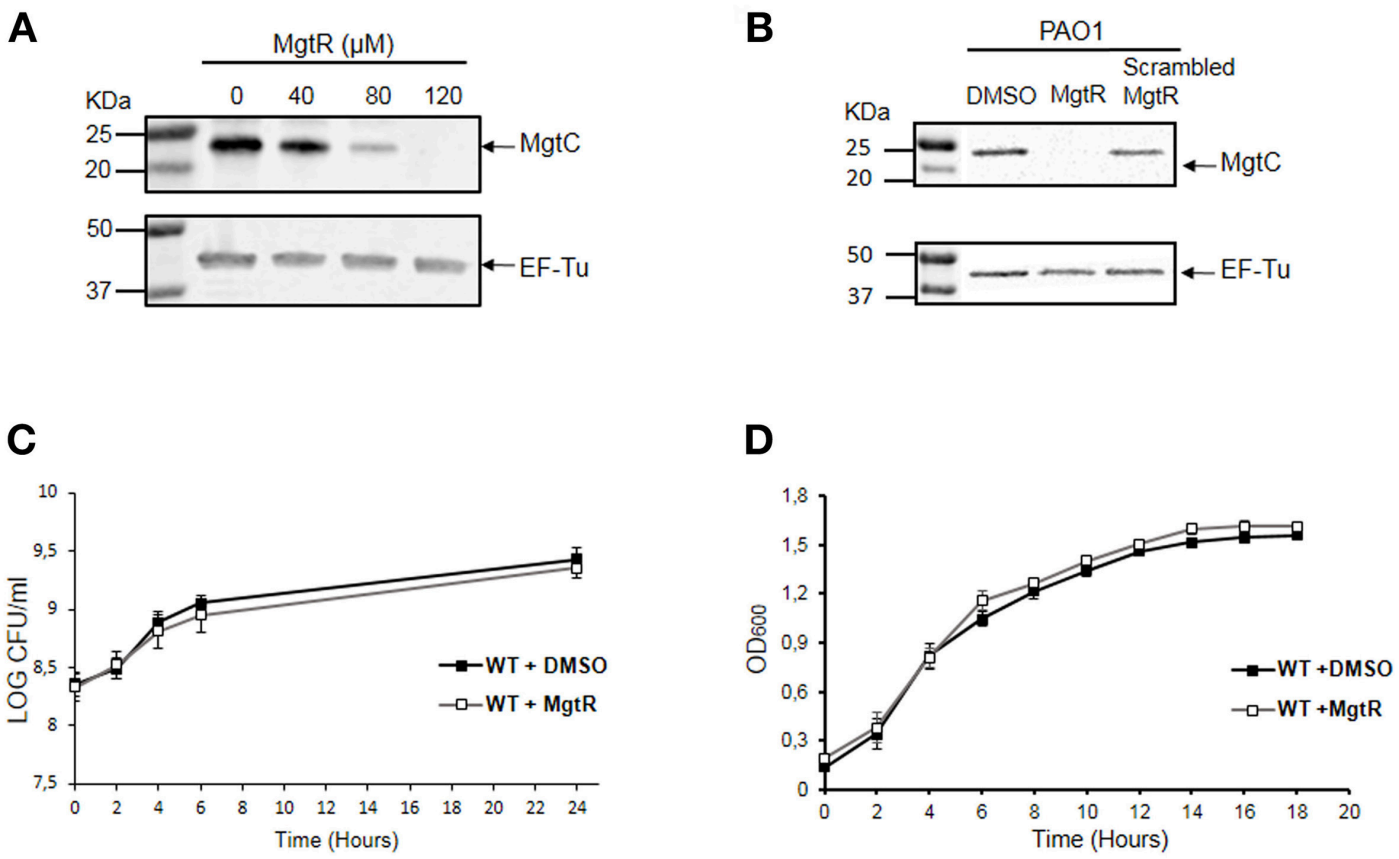
Effect of synthetic MgtR peptide on the level of MgtC protein and on *P*. *aeruginosa* growth rate. **(A)** Effect of MgtR peptide on MgtC level is evaluated on total lysates of PAO1 WT bacteria incubated during 4 h with different concentrations of MgtR peptide in NCE medium supplemented with 10 μM MgSO_4_. The membranes were probed with the anti-MgtC and anti-EF-Tu antibodies, as loading control. A representive experiment out of three independent experiments is shown. The quantified ratio PA4635/EF-Tu is 1, 0.95, 0.28, 0.01 for MgtR concentrations of 0, 40, 80, and 120 μM, respectively. **(B)** Effect of a scrambled peptide derived from MgtR on the level of MgtC protein. PAO1 WT bacteria were grown for 1.5 h in NCE medium supplemented with 10 μM MgSO_4_ before addition of 120 μM MgtR or 120 μM scrambled MgtR and were incubated further for 5 h before harvesting total lysates for blotting. A representative experiment out of three independent experiments is shown. **(C)** Effect of MgtR peptide on bacterial viability in NCE medium. PAO1 WT strain was treated with 120 μM MgtR and incubated in NCE medium supplemented with 10 μM MgSO_4_ for 24 h. At indicated time points, 10 μl of the culture was collected and diluted on LB agar plate to quantify CFUs. The results are expressed as the mean ± SD of three independent experiments. **(D)** Effect of MgtR peptide (120 μM) on the bacterial growth of PAO1 WT in LB medium. The results are expressed as the mean ± SD of three independent experiments.

To address the mechanism of action of MgtR peptide, we have conducted additional experiments upon addition of the peptide to *P. aeruginosa* bacterial cultures. Because endogenous MgtR peptide drives MgtC to degradation in *S*. Typhimurium (Alix and Blanc-Potard, 2008), we investigated the effect of synthetic MgtR peptide on the level of *P. aeruginosa* MgtC protein. Total lysates were prepared from bacteria grown in magnesium deprived medium, a condition that induces expression of *mgtC* gene (Belon et al., 2015), treated or not with peptide. Western blotting was carried out with anti-MgtC antibodies (see Materials and Methods). Hybridization with antibodies against EF-Tu were used as control (Ball et al., 2016). Addition of peptide to bacterial culture caused a decrease in the level of MgtC in a dose responsive manner, with the highest reduction at a final concentration of 120 μM, whereas the level of EF-Tu control protein remained unchanged (Figure 4A). In contrast to MgtR, the scrambled peptide had only a minor effect on the level of MgtC protein (Figure 4B).

We also monitored the effect of MgtR on bacterial viability and growth, to determine whether MgtR behaves as the known antimicrobial peptides. The effect of MgtR on the level of MgtC protein is not related to an effect on bacterial viability in NCE medium (Figure 4C), which is consistent with the constant level of EF-Tu control protein. We have also shown that addition of MgtR peptide had no effect on bacterial growth in LB medium (Figure 4D), indicating that synthetic MgtR does not have a classical antibacterial effect.

**Figure 5:**
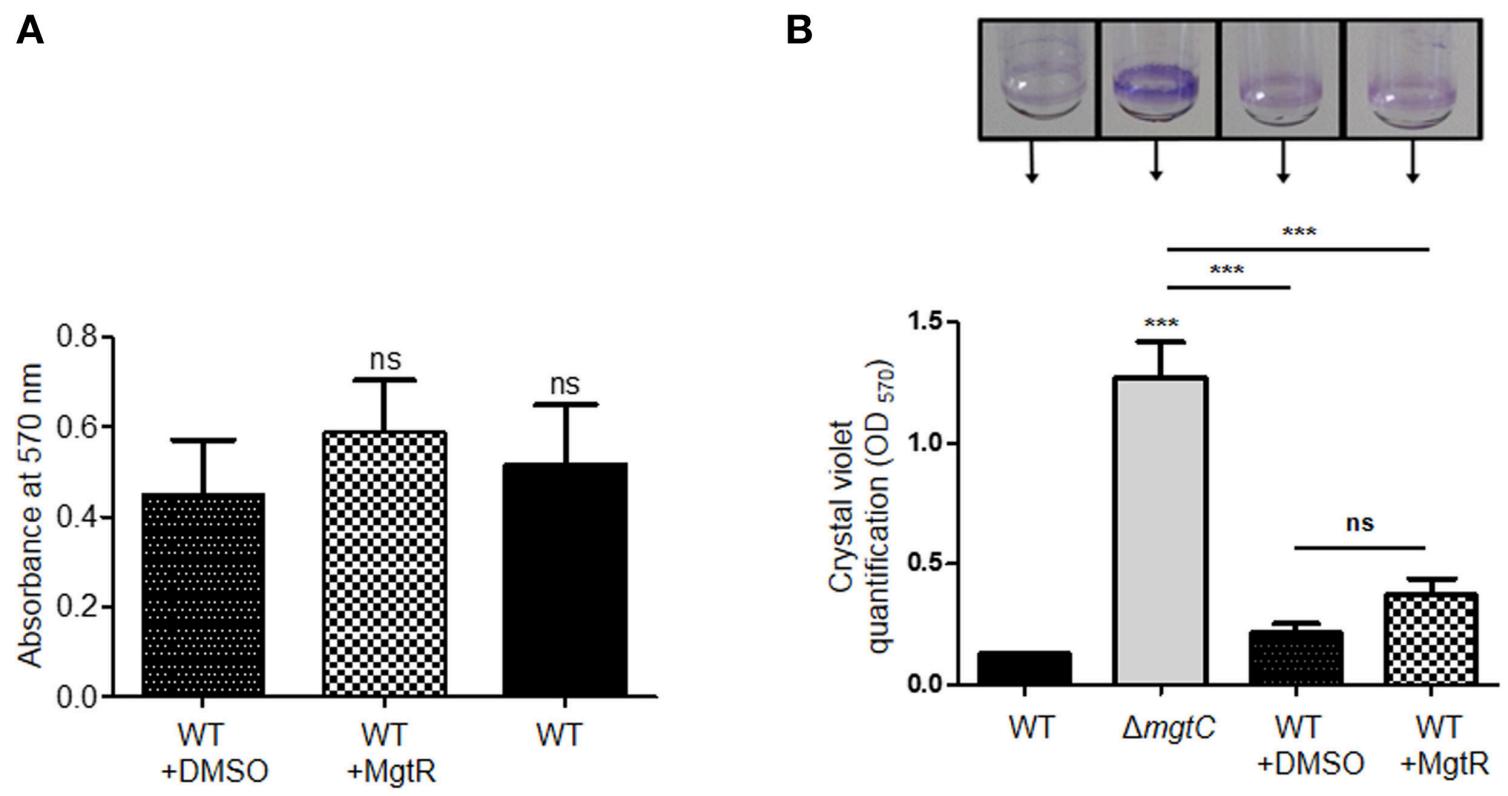
Biofilm formation in the presence of synthetic MgtR peptide. **(A)** Quantification of bacterial adherence in LB medium. PAO1 WT strain was incubated with 120 μM MgtR peptide or DMSO as control in LB and allowed to form biofilm at 28°C for 48 h in 96 well plate. Biofilm was stained with crystal violet and absorbance was measured at 570 nm. Statistical analysis was done on three independent experiments by one way ANOVA, where all samples were compared to DMSO using Dunnet's multiple comparison post-test, showing statistical significance with respect to DMSO control (ns > 0.05, non-significant). **(B)** Quantification of bacterial adherence to glass tubes in magnesium deprived medium. Adherence assay was carried out with PAO1 WT and Δ*mgtC* strains grown at 30°C for 24 h in NCE medium with 10 μM MgSO_4_ supplemented or not with 120 μM MgtR peptide. The ring on the glass tube is visualized by crystal violet staining and quantified by measuring OD_570_. Error bars correspond to standard errors (+SE) from three independent experiments and the asterisks indicate *P*-values (One-way ANOVA followed by a Bonferroni post-test, ***P < 0.001).

We further investigated the effect of MgtR on the ability of *P. aeruginosa* to form biofilm. We first tested biofilm formation of PAO1 strain grown in LB medium. Addition of MgtR did not change significantly the ability of PAO1 to form biofilm (Figure 5A). Biofilm formation was also tested in magnesium deprived medium. As shown before, increased biofilm formation is seen with the *mgtC* mutant strain comparatively to wild-type strain, but no significant increase is detected upon addition of MgtR peptide to PAO1 strain in the condition tested (Figure 5B). Moreover, bacterial viability, in the ring or in the planktonic fraction, is not significantly modified in PAO1 treated with MgtR comparatively to non-treated bacteria (Supplemental Figure S3).

Cumulatively, we have shown that, in addition to reduce intramacrophage survival, the biological action of synthetic MgtR is associated with a decreased level of MgtC protein, without any effect on growth rate. Despite the decreased level of MgtC, the exogenous peptide had no effect on biofilm formation. A similar finding was found upon endogenous production of MgtR (Supplemental Figure S4). These results may indicate that the effect of MgtR is delayed upon long term phenotype, such as biofilm. We cannot exclude a deterioration of the peptide with time. In addition, or alternatively, MgtR may also act by preventing protein-protein interaction relevant for intramacrophage survival but not for biofilm formation.

## Conclusion

Antivirulence strategies are being increasingly considered in the age of antibiotic resistance (Dickey et al., 2017) and we have previously proposed MgtC as a valuable target with a potent inhibitory peptide, MgtR. The function of MgtC has been mainly studied in *S*. Typhimurium, where it exhibits pleiotropic roles, including inhibition of F_1_Fo ATP synthase (Lee et al., 2013) and modulation of cellulose production (Pontes et al., 2015). Our results provide a link between *P. aeruginosa* MgtC and EPS production and show an implication of EPS, more specifically Psl, for the increased biofilm formation of *mgtC* mutant in magnesium deprived medium. However, EPS are not involved in the intramacrophage behavior of *P. aeruginosa mgtC* mutant, thus differing from the role reported for cellulose in the intracellular pathogen *S*. Typhimurium.

Our previous results indicated that endogenous production of MgtR lowered the intramacrophage survival of wild-type *P. aeruginosa* PAO1 strain (Belon et al., 2015). Using for the first time a synthetic MgtR peptide on PAO1 strain led to a similar effect, associated with a decrease of MgtC protein level, thus supporting the use of such peptide to target the MgtC virulence factor. MgtR did however not increase significantly biofilm formation, indicating an action towards EPS-independent phenotype rather than EPS-related phenotype. The lack of significant effect with a synthetic scrambled peptide derived from MgtR suggested that biological activity of synthetic MgtR peptide is somehow linked to sequence specificity and not to overall hydrophobicity. Hydrophobic compounds as MgtR remain difficult to handle and therefore improving peptide solubility, while keeping efficiency, will be valuable. The interest for peptides as new therapeutic molecules has recently increased (Kruger, 2017), and peptide modulators of protein-protein interaction in membranes are promising molecules (Stone and Deber, 2017). The present work thus identifies a synthetic hydrophobic peptide, issued from a natural bacterial peptide, which could limit bacterial pathogenesis and which differs from classical anti-microbial peptides (Mahlapuu et al., 2016) because of lack of effect on bacterial viability.

Further work will be required to decipher the mechanism of action of synthetic MgtR peptide. Our present results show that peptide addition is associated with a decreased level of MgtC protein but, as suggested earlier, MgtR may also prevent protein-protein interaction involving the MgtC protein (Belon et al., 2016). The lack of effect of MgtR peptide in the context of a *mgtC* mutant suggests that its biological effect is mainly related to the presence of MgtC protein. Despite this finding and the absence of noticeable growth defect upon addition of MgtR, it would be of interest to test in a more general way the impact of synthetic MgtR on membrane fluidity and integrity. Interestingly, MgtR does not appear to modulate biofilm formation, suggesting that MgtR may act more specifically during acute infection than chronic infection. Given the proof of concept provided by our results on infected cells with PAO1 strain, further work will be required to test the efficiency of such peptide on *P. aeruginosa* clinical isolates and in an animal model of infection.

## Data Availability

All datasets generated for this study are included in the manuscript and/or the Supplementary Files.

## Author Contributions

MM, PG, and A-BB-P conceived the project. MM, PN, PG, and A-BB-P designed and analyzed the experiments. MM, PN, and PG performed the experiments. BI performed construction of double mutants and EV carried out peptide synthesis and circular dichroism analysis. MM, PG, and A-BB-P wrote the paper and all authors edited and approved the manuscript.

### Conflict of Interest Statement

The authors declare that the research was conducted in the absence of any commercial or financial relationships that could be construed as a potential conflict of interest.
