# Supplementary figures and images for: Activity of a Synthetic Peptide Targeting MgtC on Pseudomonas aeruginosa Intramacrophage Survival and Biofilm Formation
Source: Front Cell Infect Microbiol. 2019 Apr 2;9:84. doi: 10.3389/fcimb.2019.00084 (PMC6454036; doi:10.3389/fcimb.2019.00084)

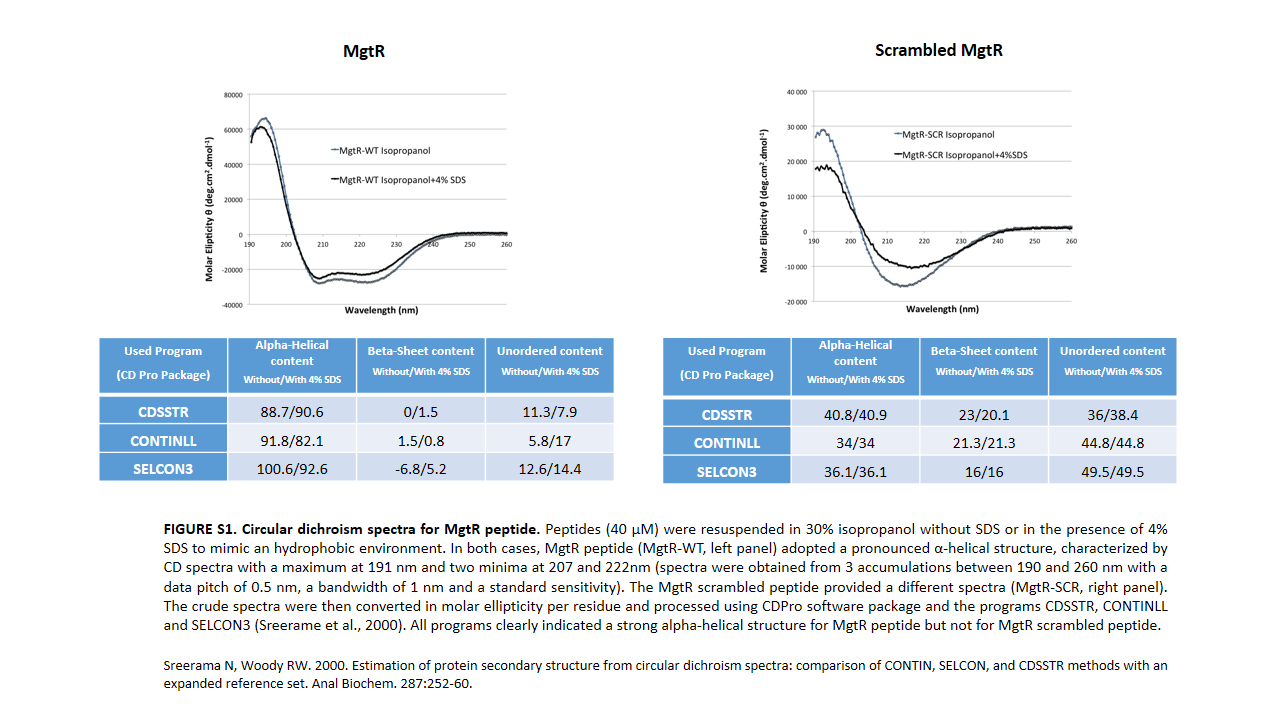

Supplement: Supplementary file 1 [file Image_1.TIF]

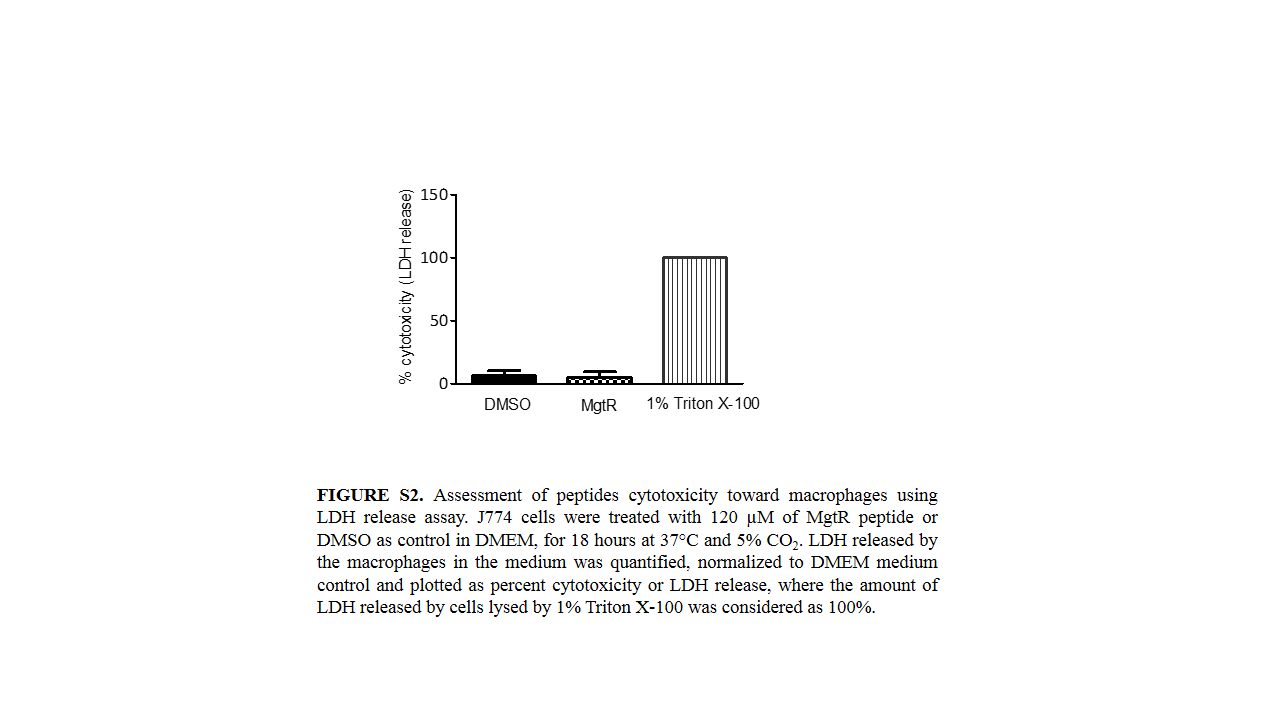

Supplement: Supplementary file 2 [file Image_2.TIF]

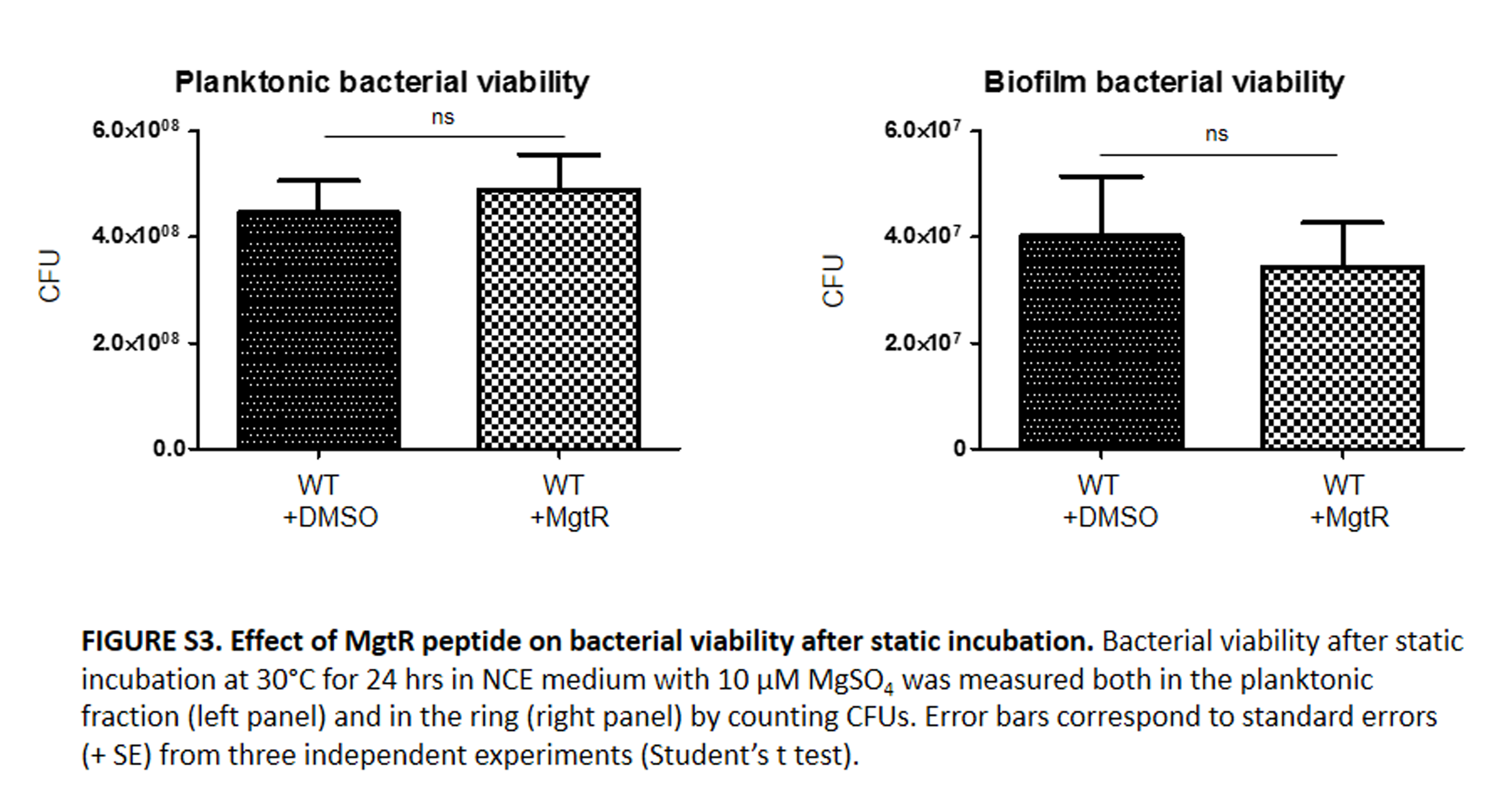

Supplement: Supplementary file 3 [file Image_3.tif]

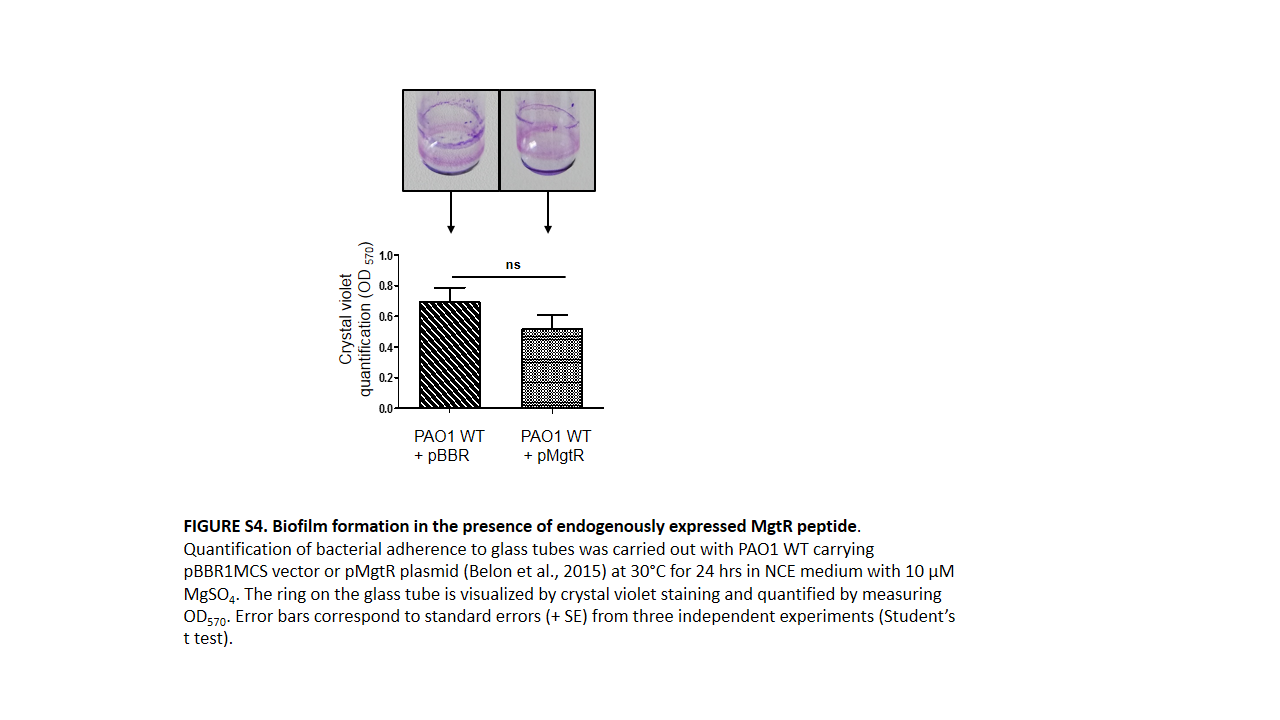

Supplement: Supplementary file 4 [file Image_4.TIF]
