# Supplementary material for: Activity of a Synthetic Peptide Targeting MgtC on Pseudomonas aeruginosa Intramacrophage Survival and Biofilm Formation
Source: Front Cell Infect Microbiol. 2019 Apr 2;9:84. doi: 10.3389/fcimb.2019.00084 (PMC6454036; doi:10.3389/fcimb.2019.00084)
Supplement: Supplementary file 5 [file Table_1.DOCX]

**SUPPLEMENTAL TABLE 1. List of primers**

| *rpoD*-RT-fwd | 5’-GGGCGAAGAAGGAAATGGTC-3’ |
| --- | --- |
| *rpoD*-RT-rev | 5’-CAGGTGGCGTAGGTGGAGAA-3’ |
| *pslA*-RT-fwd | 5’-GTTTCCCTACCTCAGCAGCA-3’ |
| *pslA*-RT-rev | 5’-CGGATGTCGTGGTTGCGTA-3’ |
| *pelA*-RT-fwd | 5’-CCTTCAGCCATCCGTTCTTC-3’ |
| *pelA*-RT-rev | 5’-TCGCGTACGAAGTCGACCTT -3’ |
| *algE*-RT-fwd | 5’-GTTTCAGCGAATACCGCACC-3’ |
| *algE*-RT-rev | 5’-AGCTGGCCGGTATAGGTCTT-3’ |
| PA4635 Cter F | 5’-CGGGATCCGCCAGCGAGGCCGAGCAAC-3’ |
| PA4635 Cter R | 5’-CCCAAGCTTTCAGTCGGCCGCCAGTTC-3’ |
